# Exploring Vaccine Hesitancy, Structural Barriers, and Trust in Vaccine Information Among Populations Living in the Rural Southern United States

**DOI:** 10.3390/vaccines13070699

**Published:** 2025-06-27

**Authors:** Alice R. Richman, Abby J. Schwartz, Sarah B. Maness, Leslie Sanchez, Essie Torres

**Affiliations:** 1 Department of Health Education and Promotion, College of Health and Human Performance, East Carolina University, 300 Curry Court, Greenville, NC 27858, USA; osoriopascuall23@ecu.edu; 2 School of Social Work, College of Health and Human Performance, East Carolina University, 114 Rivers, Greenville, NC 27858, USA; schwartza15@ecu.edu; 3 Office of the Vice Chancellor for Research, 312 South Building, University of North Carolina at Chapel Hill, Chapel Hill, NC 27599, USA; essie.torres@unc.edu

**Keywords:** vaccine equity, vaccine hesitancy, vaccine information, rural health

## Abstract

Introduction: In the United States, vaccine hesitancy is higher among rural and racially and ethnically diverse communities, and messaging from trusted individuals may increase vaccine acceptance. The purpose of this study is to understand vaccine hesitancy, messaging from trusted individuals, and vaccine acceptance strategies among racially and ethnically diverse, medically underserved rural populations. Methods: The researchers conducted 12 in-person focus groups, each consisting of 5 to 12 participants, with community members and trusted leaders from three rural counties in Eastern North Carolina (*n* = 119). Thematic analysis was used to synthesize insights from the discussions, allowing for the identification of recurring patterns and community-specific considerations regarding vaccine perceptions and messaging. Results: The researchers identified seven key themes within the primary focus areas of the study: factors influencing vaccine hesitancy, messaging from trusted individuals, and strategies to improve vaccine acceptance. Participants reported differences in trust based on how long a vaccine has been available, concerns about becoming sick after a vaccine, seeing the symptoms of vaccine-preventable diseases, and misinformation on social media. Overall, participants reported trust in messages from medical providers. Trusted leaders advised people to conduct their own research on vaccines when determining whether to receive vaccinations. Lastly, social determinants such as cost, education, and transportation were identified as key barriers to vaccination. Conclusions: Our findings indicate that medical providers are trusted messengers for vaccine information and the promotion of vaccine uptake. However, distrust linked to fear, misinformation, and structural barriers persist. Public health efforts to increase vaccination confidence among rural, racially and ethnically diverse populations in the United States Southeast should address these factors in future vaccine interventions and educational efforts.

## 1. Introduction

Vaccination is one of the greatest public health achievements, resulting in the eradication of smallpox globally and the control of diseases including measles, mumps, rubella, diphtheria, pertussis, tetanus, yellow fever, and polio in the United States [1,2,3]. However, vaccine uptake levels must be high to be successful in decreasing infectious disease [4]. In the U.S., three quarters of adults are missing one or more routinely recommended vaccinations [5]. Lower uptake has led to a greater number of recent outbreaks of vaccine-preventable disease [6,7,8,9]. In line with lower vaccine uptake, there are increasing trends of vaccine hesitancy and perceptions that vaccines are not safe [10].

It is important to understand why people choose to reject vaccines when they are safe, easily accessible, and often free. Vaccine hesitancy is complex and varies across contexts and populations. Contributors to vaccine hesitancy include both individual and group factors, such as personal perceptions and social and peer influences, as well as contextual factors, which can be historic, sociocultural, environmental, institutional, economic, or political [11]. Hesitancy can also be vaccine-specific, meaning that the risk or benefit of the exact vaccine in question can be influenced by aspects like newness or cost. Additional factors that are key in vaccine decision-making include trust in advice given by healthcare providers, trust in mainstream medicine, and general views toward health [12]. Ultimately, vaccine hesitancy has been shown to result in vaccine delay or refusal [13,14]. Vaccine delay or refusal place both the individual’s health and the health of those around them at risk through community transmission [15].

Disparities in vaccine uptake are higher in rural areas for routine vaccines [16,17,18]. Adolescents from rural areas receive fewer HPV and meningococcal vaccines [19], and COVID-19 vaccination is lower in rural areas for all sex and age groups [20]. Racial and ethnic disparities also exist in vaccine uptake. Black and Hispanic Americans are less likely to receive influenza or pneumococcal vaccines than their non-Hispanic White counterparts in the same age groups [21,22]. These data are notable considering that rural and racial/ethnic minority groups also experience an increased disease burden for many diseases with available vaccines. Rural communities experience higher hospitalization rates for immunizable conditions [23]. Flu hospitalization rates were nearly 80% higher among Black adults from 2009 to 2022 and 20% higher among Hispanic adults than White adults [24]. Racial and ethnic minorities also experienced disproportionate disease severity regarding COVID-19 in comparison with White adults [25]. Given the existing demographic and geographic vaccination disparities, it is important to understand vaccine hesitancy among racially and ethnically diverse populations living in rural areas.

Individuals with high levels of vaccine hesitancy often also have high levels of mistrust [26]. Trusted sources of information may reduce vaccine hesitancy [27]. However, who community members regard as trusted individuals may vary across populations [28]. Identifying trusted sources and the ways in which they relay vaccine messages is imperative to reduce disparities in vaccine-hesitant diverse populations.

Determinants of vaccine hesitancy among marginalized communities are not well understood. This study builds on previous work indicating a need for clear, accurate, and trusted information about both COVID-19 vaccines and a wider range of more recently developed vaccines [29,30]. The purpose of this study is to understand vaccine hesitancy among racially and ethnically diverse and medically underserved rural populations, to assess vaccine messaging from trusted individuals (e.g., clergy/church leaders, community health workers), and to identify strategies to improve vaccine acceptance among these populations.

## 2. Materials and Methods

### 2.1. Study Design, Participants, and Setting

The researchers employed a descriptive phenomenological approach to guide the study [31]. This approach allowed for an in-depth understanding and description of the lived experiences of community leaders and members living in the rural southeastern part of the United States. Participants discussed their lived experiences in relation to their experiences with vaccines (trust of vaccines, vaccine messaging, and barriers to/facilitators of vaccine administration). Using phenomenology to orient the research required the researchers to approach the topic with an open mind, free from external interpretations, and to also reflect on how the researchers’ own beliefs and experiences might affect the research and findings [31].

The researchers conducted 12 in-person focus groups, each consisting of 5 to 12 participants, with community members and trusted leaders from three rural counties in Eastern North Carolina (ENC). Participants included both English- and Spanish-speaking individuals. Each focus group session lasted approximately one hour. As an incentive for participation, individuals received a USD 50 VISA gift card.

To facilitate discussions, the researchers developed structured topical focus group guides tailored to measure key themes, including barriers to vaccination, perceptions of vaccine-related messaging, general attitudes toward vaccines, and preferred methods for receiving vaccine-related communication. The focus groups were conducted in community settings that were familiar and accessible to participants, such as a community center or a private room in a local restaurant, ensuring a comfortable environment for open dialog. All study procedures received approval from the [blinded for review], ensuring compliance with ethical research guidelines.

### 2.2. Measures

#### 2.2.1. Focus Group Guides

Two separate but thematically aligned topical guides were developed: one for community members and one for trusted leaders. Each guide included an introductory section outlining the purpose of the study, the roles of facilitators, informed consent procedures, participation guidelines, and an icebreaker activity to build rapport among participants. A sample guide can be found in Appendix A.

For focus groups with community members, discussions were structured into three main sections. The general vaccine discussion focused on participants’ understanding of vaccines, factors influencing trust or distrust in vaccines, and the broader community’s perceptions of vaccinations. The vaccine messaging section explored how participants perceived vaccine-related messages from government agencies, healthcare providers, and trusted leaders. This section also addressed the impact of these messages on attitudes toward vaccination and identified preferred messaging strategies that would be most effective within their communities. Finally, the barriers to vaccination section examined obstacles such as logistical challenges, accessibility, and personal or cultural concerns. Participants also shared firsthand experiences with barriers that had prevented them or their friends or family members from receiving vaccinations.

**Figure 1:**
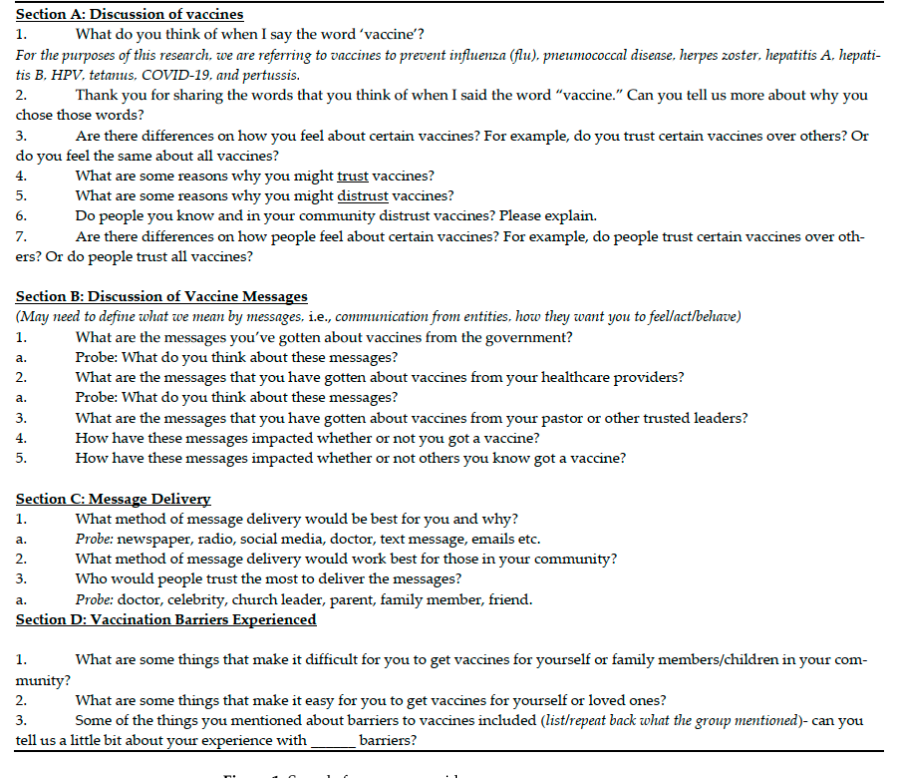
Sample focus group guide.

For focus groups with trusted leaders, the topical guide followed a similar structure but was adapted to capture perspectives unique to their roles in the community. Additional questions included how their communities perceived vaccines, the guidance that they provided to community members regarding vaccination, and the most effective communication strategies for promoting vaccine uptake within their communities. A sample of the instruments used to guide the discussions can be found in Figure 1.

#### 2.2.2. Demographic and Attitudinal Survey

Before participating in the focus group discussion, all individuals were provided with a paper and pencil survey that asked about their demographic background and attitudes toward vaccines and trust. Demographic survey questions consisted of five questions related to job/profession, years in their role (if a community leader participant), sex, race/ethnicity, and age. Additionally, participants were asked about their concerns about vaccination and their level of trust in the healthcare system, healthcare providers, community leaders, church/spiritual leaders, the government, and the media. Attitudinal survey measures were used from validated items tested in previous research [29]. This survey provided a contextual background for the participants’ characteristics and their pre-existing vaccine-related beliefs, which contributed to a more nuanced interpretation of the focus group discussions. This survey was available in English and Spanish (see Appendix A for a sample survey).

#### 2.2.3. Data Collection Procedures

Recruitment efforts relied on established community connections to ensure participant diversity and engagement. A.R. and A.S. moderated all focus groups conducted in this study. They had no prior relationships with the participants. For recruitment, community contacts implemented a snowball sampling approach to engage potential participants. Trusted leaders were identified through established relationships with religious, cultural, and health-related organizations in ENC and recruited during each organization’s leadership meetings. Examples of trusted leaders included pastors, county commissioners, public school teachers, healthcare professionals, and community health workers. Trusted community members facilitated recruitment through word-of-mouth outreach and the distribution of flyers in highly frequented locations, including churches, laundromats, and other community gathering spots. These individuals also personally invited community members to participate. The research staff conducting the focus groups had limited involvement in recruitment to minimize potential bias. Data saturation was met as no new information concerning perceptions or experiences was shared as the focus groups progressed [32].

For flyer-based recruitment, interested individuals were instructed to call a designated phone number for additional information and to register for a scheduled focus group. The project manager recorded participant details and conducted reminder phone calls before each session. Some registrants opted out before the scheduled date due to scheduling conflicts or waning interest. In several cases, individuals inquired about bringing friends or family members to participate. When this occurred, the project manager ensured that the referred individuals were informed about the study’s purpose before confirming their participation.

### 2.3. Qualitative Analyses

All focus group sessions were audio-recorded and transcribed verbatim and lasted approximately 90 to 120 min. Focus group leaders took field notes after each group meeting to reflect on any preconceived ideas, background, and/or experiences concerning the research topic that could impact the interpretation process. Study staff reviewed transcripts for accuracy before analysis. To ensure consistency in data interpretation, transcripts from the community member and trusted leader focus groups were analyzed together. Two research team members independently reviewed all transcripts and collaboratively developed a structured codebook based on emergent themes. Data were coded using the NVivo qualitative analysis software. One researcher conducted the primary coding of all transcripts, while a second coder analyzed a randomly selected 40% subset to assess reliability. The coders engaged in iterative discussions to resolve discrepancies and refine the thematic categories. Additionally, the codebook and key themes identified through coding were reviewed with the study team to ensure consistency across the coding schema.

Thematic analysis was used to synthesize insights from the discussions, allowing for the identification of recurring patterns and community-specific considerations regarding vaccine perceptions and messaging [33]. We conducted a qualitative thematic analysis using the NVivo software to organize and code the focus group data. Two independent coders (S.M. and L.S.) participated in the analysis. The process began with the coders familiarizing themselves with the transcripts, followed by the development of a structed codebook based on both the interview questions and the emerging themes from the data. The codebook was refined iteratively and used consistently across all transcripts. Discrepancies in coding were discussed and resolved by consensus to ensure reliability in theme identification. The final codebook included code names, descriptions, and the focus group questions that they were derived from. The codebook also included high-level themes and sub-themes developed during the analytic process.

## 3. Results

Overall, 119 individuals participated in the focus group discussions, including 91 community members and 28 trusted leaders. Most community member participants (84%) and leaders (63%) identified as Black or African American. Additionally, 18% of community members and 33% of leaders identified as having a Hispanic ethnicity. The majority of both the community members (70%) and leaders (60%) were female. Only 11% of community members reported attaining a college degree or higher, while 65% of leaders had a bachelor’s degree or higher. Community members had a mean age of 60 (SD 15.2), while the leaders’ mean age was 50 (SD 26.1). Most community members had a family income of less than USD 30,000 (53%). Additional demographic information for the focus group participants can be found in Table 1.

Responses to the attitudinal survey items indicated that participants had the highest levels of trust for healthcare providers, church/spiritual leaders, and the healthcare system. Participants reported moderate trust in community leaders and the least trust in the media and government (Table 2). When asked about concerns about receiving vaccines, the most reported concerns were about side effects (47%) and worries about selecting between many available options (46%) (Table 3). Over a third of the participants had no concerns about receiving vaccines (*n* = 37%).

The researchers identified seven key themes from the transcript analysis within the three primary focus areas of the study: understanding vaccine hesitancy, messaging from trusted individuals, and strategies to improve vaccine acceptance. Community member and leader data were analyzed together, as the coding revealed that the qualitative responses were not dissimilar enough to warrant separate analyses. The table in Appendix B provides illustrative quotes across roles and themes, further demonstrating the similarity in the responses from community leaders and community members and substantiating the need for combined analyses.

### 3.1. Primary Focus 1: Understanding Vaccine Hesitancy Among Racially and Ethnically Diverse and Medically Underserved Rural Populations

#### 3.1.1. Theme 1: Differences in Trust Based on How Long a Vaccine Has Been Available

When stating reasons to distrust vaccines, participants often felt that newer vaccines were less credible. Participants were concerned that, with new vaccines, there is uncertainty regarding whether they work, confusion about why there are so many so rapidly (e.g., COVID-19 boosters), and why only certain vaccines can be created so quickly.

*“I’m trying to figure out why they rushed when there’s other stuff out there killing people like cancer…I mean you get the COVID vaccine approved in less than a year, but you can’t cure nothing else that’s been out here for years.”*

(Focus Group 1, Community Member)

When asked about the reasons that they trusted vaccines, many participants said that they trusted the vaccines that they had to receive to attend school as a child and because they had seen over the years that they had worked. However, when asked if they felt differently about various types of vaccines, many participants were skeptical of new vaccines since they were developed and disseminated very quickly and were concerned that they did not work.

*“Like [participant name] said about the shots we got when we were kids, I always thought they were good because you know I got those as a kid and … I’m good. So, it was no question when it was time for my kids to get ‘em. When it was time for my kids to get those shots…. No problem. But like she said when they start introducing new stuff. Um, I know we’re not solely talking about COVID today but, just you know when COVID came out, the vaccines came out so quick [snaps]. And so many different ones…. Yeah, I don’t know if I trust that.”*

(Focus Group 2, Community Member)

#### 3.1.2. Theme 2: Distrust Influenced by Misinformation, Often Spread Through Social Media

The fears about vaccines that participants shared or mentioned hearing from others were often related to misinformation on social media. Participants specifically mentioned young people as being particularly influenced by social media and believing information that may not be credible.

*“I definitely see distrust … because let’s say, someone’s aunt or the friend of someone on Facebook or social media posts like, ’the vaccines are like harming people and causing these other illnesses’. And that could be like completely unrelated to the vaccines, and they’re just attaching it to whatever was going on with them... And they tell their other friends, their husbands, and they start just associating that with negative connotations and with vaccines overall. A lot of like distrust based on misinformation.”*

(Focus Group 11, Leader)

*“One thing that I’ve noticed… a lot of the young people that are taking social media at face value … a lot of the younger generation right now, they are perpetual rebelers [sic]. They want something to rebel against. And if you present something to them and you’re telling them five reasons why it will help, they will give you six reasons why it won’t.”*

(Focus Group 9, Leader)

Some participants reported the view that vaccines are a means of control by the government. Five focus groups, including among trusted leaders, mentioned the idea that the COVID-19 vaccine involved inserting a chip into the recipient that enabled the government to track them. These comments were not always framed to indicate that the participants themselves believed this but that they had heard it from someone else.

*“Well, you know, like a chip. Not like a chip but a form of … like radiation, something inside your body… with certain strains it will appear up…like we go to the hospital, and we got to take a CAT scan. They give us something to drink and then they run it through a machine, and they can detect certain things. So, if they use a vaccine to detect, you know, like look at from a certain strain, like okay, there’s a vaccine.”*

(Focus Group 1, Community Member)

Participants generally did not prefer the government as a source for vaccine messaging. Some participants felt that messages from the government provided good information, while others had concerns about being controlled, tracked, or lied to. Ideas specific to COVID-19 included skepticism about the politicization of vaccines and false information from government sources.

*“Yeah, when the vaccine first started, the first go-round, well, ‘if you take it you’re going to be a zombie.’ I mean this was people in their forties believing this stuff. The government controlling your mind and knowing where you are.”*

(Focus Group 10, Leader)

*“Going back to the 50/50 because some people trust the CDC. But then with social media, the other half doesn’t trust them … people also see it kind of like in the way that they see like a parent like, ‘oh, they’re just bugging me to manage me. It’s just a form of authority that I don’t want to listen to.’”*

(Focus Group 11, Leader)

#### 3.1.3. Theme 3: Concern About Becoming Sick After a Vaccine

Many participants mentioned distrust in vaccines because either they or their family members had become sick after taking a vaccine. The specific vaccines most mentioned in relation to becoming sick after receiving them were the COVID-19 and flu vaccines.

*“Um, I do not take those… I took the flu vaccine for five years straight I got the flu. And when I stopped taking them, I haven’t had the flu since then. Had a little slight cold or whatever but I never had the flu for it to be aching all for about three or four days.”*

(Focus Group 3, Community Member)

When asked if they felt differently about different vaccines, several participants stated that they did not receive the flu vaccine because it made them sick. Five participants directly stated that the flu vaccine causes the flu.

*“When I went to the doctor, he asked me about the flu shot and the COVID. And I told him I had the COVID but I’m not taking the flu because every time my dad would take the flu shot, he would get the flu.”*

(Focus Group 6, Community Member)

#### 3.1.4. Theme 4: Trusting Vaccines Due to Seeing the Impact of Not Taking Them

Participants often stated that they personally trusted vaccines because they had been shown to work and kept them alive when they saw others become sick or die. Participants who trusted vaccines also reported that vaccines kept them healthy and saw them as positive.

*“… back when we were coming up, I didn’t hear anything about HPV. So, you know when I got older and started hearing about it, I’m like, ‘oh wow what’s that?’ And from a personal experience I know a family member that passed from it. So, that’s why I got my kids vaccinated for it because that’s when I started hearing about when it affected my family… you know it’s for males also. My son is also vaccinated from it. So yeah, because of that personal experience I had, I definitely said, ‘ok yeah, we’re gonna get it.’…new stuff I’m kinda skeptical, about … you know just gotta weigh your pros and your cons. Gotta weigh your options.”*

(Focus Group 2, Community Member)

Some participants described a comparison of their own experiences pre- and post-vaccination—for example, noticing how much sicker they were when they had COVID-19 before being vaccinated versus a milder form afterwards.

*“I got COVID and then I got vaccinated, right? I caught it again after I got vaccinated. The reason I went and got the booster, although it wasn’t mandatory for my job, is because I saw the difference in when I didn’t have it, when I wasn’t vaccinated. I thought I was about to die.”*

(Focus Group 1, Community Member)

### 3.2. Primary Focus 2: Assessing Vaccine Messaging from Trusted Individuals

#### 3.2.1. Theme 5: Trusted Leaders Advise People to Conduct Their Own Research on Vaccines

When asked what they would advise community members to do regarding vaccination, trusted leaders advised people to conduct their own research rather than listen to anyone else. Every group specifically used the term “do your own research”.

*“I would definitely advise anyone to do their own research…. I could tell you anything, your doctor could tell you anything, your pastor could tell you anything. But, if you don’t look it up for yourself… you will never know. So, I always advise people, ‘do your own research.’”*

(Focus Group 9, Leader)

When trusted leaders gave advice for people to conduct their own research, they often mentioned using valid websites or trusted sources. Examples of trusted sources included the health department or someone from the community who had already taken the vaccine.

*“First, I would tell them to learn all they can about the disease; what it does, what would happen to you if you had the disease, and to be specific. Look at it from your age group. So, someone getting chickenpox at five years old it’s going to be vastly different than someone getting chickenpox [at] my age. Or, like mumps. I’ve never had them so when I was working in the hospital one time, I had to go home. There was a gentleman on the floor with mumps. The director of nursing, she was like, ‘we have mumps’ and she said, ‘I’m sure everybody here had it’ I said, ‘I’ve never had mumps.’ She said, ‘you may leave.’ Mumps in a child is fine. If you are of childbearing age, mumps can make him sterile. Yes, that’s why it’s dangerous in men. So, find out how that affects your group. Go to valid websites.”*

(Focus Group 10, Leader)

#### 3.2.2. Theme 6: Trust in Messages from Medical Providers

Participants viewed their doctors telling them to take a vaccine as one of the main reasons that they trusted vaccines.

*“I believe that we all have a trusted doctor. And we go with the opinion of that doctor. Why? Because we trust him and maybe he’s been seeing us, the family, everyone for many years, and maybe we’ve seen the sequence that has been correct, so we say, ‘well, yes.’”*

(Focus Group 8, Community Member)

Participants also reported a clear preference for vaccine messaging to be delivered by a medical professional. Although responses for the method of delivery varied widely (church, radio, email, senior center, etc.), there was largely a consensus on doctors as the preference regarding who should deliver the message.

*“The doctor would be more up to date than the media would. If there was word out there, I think the healthcare provider would know because they’re familiar with things. They’re getting more information about it all the time. To me it was just get a shot, boom, boom.”*

(Focus Group 6, Community Member)

Although most participants reported trusting vaccine messaging from healthcare providers, several participants actively stated that they did not trust doctors and that doctors pushed people to be vaccinated.

*“Because I had one doctor that was trying to get me to get it because of my health situation and I had to tell him to stick to his topic of treating me, for what he treated me for. I mean don’t push me to get COVID shots. It ain’t going to cure me.”*

(Focus Group 1, Community Member)

#### 3.2.3. Theme 7: Social Determinant Barriers to Vaccination (Cost, Education, Transportation)

The major reported structural barriers to vaccination were a lack of transportation, the high cost, and a lack of education to make informed decisions about whether to be vaccinated. Nearly every focus group mentioned transportation as an issue. Unawareness of aspects like the actual cost of vaccines or the availability of payment based on a sliding scale was another barrier mentioned by both community members and leaders. Participants mentioned that vaccines could be very expensive without insurance. Participants also reported instances of vaccines not being available or difficulties in navigating the process of finding and paying for them.

*“Transportation also is definitely a barrier, because I work [in] transportation, and if the referrals of people they can’t get to their appointments because they don’t have a way, or they don’t have the gas… You know our location itself… they’re putting up doctor’s offices in smaller areas so it’s a little easier to get there but you know, transportation is a barrier for a lot of people. The elderly um, it’s just bad for them.”*

(Focus Group 2, Community Member)

*“… a vaccination campaign, for example. If a campaign like that were to happen here, I assure you that there would be a crowd because many people want it and can’t afford that vaccine. So, organizing campaigns…with specific vaccines… what people need most and don’t have access to, either due to the price or because they don’t have access to health insurance, right? So, health authorities should organize these vaccination campaigns … for people in rural areas. But what happens?… The quota is very limited. If people go, they’re told ‘no, you’re not a farm worker’ or ‘you don’t live here.’ They ask for proof of residence and ‘no, it doesn’t match,’ so you’re turned away.”*

(Focus Group 8, Community Member)

When asked about facilitators of vaccination, participants often mentioned factors that would make it easier to receive vaccines but were not currently in place. Participants stated that they would like to see mobile units or home visits for vaccination but that many ended after the COVID-19 pandemic or did not provide vaccinations. Having free or low-cost options and public campaigns to increase awareness and education were suggestions raised to facilitate vaccination.

*“I wonder if it would be good like for people who are house bound, have someone to come in. I know like with insurance companies with my sister they had someone come in and do these tests and everything. If they had someone like that who could come in and give vaccinations, someone that you trust, not just anybody coming in.”*

(Focus Group 1, Community Member)

## 4. Discussion

Overall, our findings indicate varied factors influencing vaccine hesitancy among this racially and ethnically diverse and medically underserved rural population. Many felt that seeing people become sick from vaccine-preventable diseases and being advised by their doctors led to greater trust in vaccine efficacy; however, skepticism over new vaccines, concerns about becoming sick after vaccination, and misinformation on social media continue to impact vaccine hesitancy. Quantitative attitudinal data supported the qualitative findings, indicating high trust in healthcare providers and worries about becoming sick from vaccines. The community members’ and leaders’ qualitative responses were more similar than expected, leading to the study team’s decision to combine these responses for the qualitative analysis. The leader sample had markedly higher levels of formal education, which has been linked to reduced vaccine hesitancy, yet we found hesitancy and reports of misinformation in both groups [34].

Our results showed differences in trust based on how long a vaccine has been available. A scoping review of studies published during the COVID-19 pandemic found that many people’s reason for refusing vaccination included thinking that a vaccine produced in a rush is too dangerous [35]. One study, specifically including Hispanic populations in rural South Carolina who had not been vaccinated for COVID-19, found that participants feared that the vaccine’s development had been rushed and that being vaccinated might cause them to become sick and miss work [36]. An additional qualitative study conducted in ENC found that participants distrusted the COVID-19 vaccine because they felt that it was developed too soon and were concerned about side effects [30]. These findings align with our study results indicating that there is less trust in newer vaccines and concerns about becoming sick after vaccination. The skepticism about how vaccines are able to be produced so quickly indicates a need for public health education that focuses on how vaccines are created.

Our study’s findings on vaccine misinformation from the media align with previous work indicating that the internet and social media are major drivers of inaccurate data or misinformation about vaccines [37]. Additionally, research indicates that social media users are more likely to believe information from non-scientific or pseudoscientific influencers than scientific information [38]. This may reflect larger contextual factors, like the wider decline in trust in expertise and authority [39]. The participants often referred to younger people as the main group who believe information that they read on social media. A study examining rural and urban young adult vaccine hesitancy for the COVID-19 vaccine found that rural participants had 40% lower odds of intending to receive the COVID-19 vaccine [40].

When participants discussed becoming sick after a vaccine, it was not always clear whether they were describing side effects from the vaccine or later becoming sick with the disease that the vaccine was intended to prevent. For example, multiple people stated that they or their family members contracted the flu from the flu vaccine, but this is not medically possible [41]. This indicates a need for further education differentiating between reactions after a vaccine and contracting the disease. One study indicated that people who did not receive the flu shot and later contracted the flu had a higher likelihood of being vaccinated the next year. However, those who received the flu shot and still later contracted the flu were less likely to be vaccinated the next year [42]. Providing nuanced information indicating that some vaccines do not prevent all strains, like the flu, or may result in a less severe case of the disease rather than no disease (e.g., COVID-19), could reduce fears that vaccines do not work.

Alternatively, participants voiced feelings of trust in vaccines after seeing the impacts that diseases have on those who are unvaccinated versus those who have been vaccinated. At times, this included participants’ families experiencing disease or even experiencing disease themselves and then deciding to be vaccinated. Seeing people that one knows experience the negative effects of diseases may increase the perceived risk and severity. This was demonstrated in a previous study using the Health Belief Model to predict vaccine intentions related to COVID-19 [43].

When assessing vaccine messaging from trusted leaders, the advice from the leader focus groups was largely for community members to conduct their own research on vaccines. A recent study examining how the slogan “do your own research” has been used online (public posts on Instagram and Facebook) found that this slogan is often used by anti-vaccine activists but also can be used to activate civic responsibility [44]. These types of messages promote individual responsibility for navigating online information and may reflect wider societal distrust of institutions. Notably, trusted leaders provide this suggestion, rather than to speak with a physician, while participants themselves preferred to receive messaging from healthcare providers.

Public health efforts can work to counteract anti-science narratives through repairing trust in vaccines and in the government and other groups. These efforts can work through community-level strategies like educational campaigns and community health worker-delivered interventions and through provider-level strategies such as provider vaccine recommendation interventions. Community-level strategies should promote accurate vaccine information and should highlight the importance of talking to a trusted person like a healthcare provider for guidance and information about age-appropriate vaccines. Educational campaigns and community health worker interventions should promulgate the idea to “do your own research” but also equip the community with the skills to identify the differences between reputable and biased vaccine informational sources. Provider vaccine recommendation interventions should work with healthcare providers to ensure that they provide strong, clear vaccine recommendations to their patients to increase the uptake of vaccines. Providers should explain the benefits of vaccines while addressing any patient concerns. It is interesting that, while this study utilized a trusted messenger approach, community members largely wished to hear vaccine messaging from medical providers. Previous research among both rural populations and racial and ethnic minorities has supported the notion of strengthening vaccine campaigns by including messaging from trusted individuals [45,46]. This is consistent with the present research team’s previous work showing that the preferred COVID-19 vaccine messaging was through word of mouth and personal testimony from trusted individuals [30]. However, a narrative review of HPV vaccine trust among racial and ethnic minorities in the U.S. showed high levels of trust in doctors and healthcare providers but less trust in pharmaceutical companies and government agencies [47]. As healthcare providers are a trusted source for vaccine information, it is important that providers have training to consistently provide clear and accurate recommendations. Previous work found that providers outside of the U.S. Southeast had 5.2 higher odds of recommending COVID-19 vaccination, indicating a need to improve provider vaccine recommendations in the Southeast [29].

Lastly, the identification of strategies to improve vaccine acceptance should consider social determinant barriers to vaccination. Our findings included barriers such as cost, education, and transportation, which are consistent with the literature. A qualitative study on barriers to HPV vaccination in rural Georgia also found transportation to be a barrier to vaccination [48]. In rural, medically underserved locations, the distance to a medical center can be large. The distance to providers was also found as a barrier in a systematic review of early childhood immunization in the rural U.S. [49]. Another study examining vaccination among older adults found associations between cost and vaccination, as well as additional barriers for less educated, minority, and rural participants [50]. The majority of participants in this study reported a low family income, which aligns with the qualitative finding that cost is a barrier. Access issues are often focused on not bringing services to the community and people needing to find out how to access the service, in terms of both transportation and payment, as well as other restrictions, like residential requirements. Even if negative perceptions about vaccinations improve, if structural barriers persist, they will impede optimal vaccine uptake.

This study is not without limitations. Participants willing to participate in a focus group about vaccines may have different views compared to those who prefer not to share their opinions. The sample for this study was self-selected and consisted of mostly Black/African American, female, older adults with a lower income, which limits the generalizability of the results and could have introduced selection bias. The demographic survey did not ask about the jobs or roles of trusted leaders (i.e., pastor, community health worker, government worker, etc.), so we were unable to include this information in our interpretation of the data. Data on the professions of the community leaders were based on the memory of the focus group moderators. The demographic survey was completed in person prior to the focus group discussion to ensure a better response rate, but not all participants fully completed the demographic survey. In addition, although we captured participants’ levels of trust in organizations (e.g., healthcare system, government, media) (Table 2), understanding the difference between “somewhat” trusting and “not at all” trusting, and its association with vaccine decisions, would have been important. Thus, future research concerning vaccine hesitancy should disentangle the difference in the levels of trust and their impacts on vaccination uptake. Our findings indicate that medical providers are key to trust in vaccines and a desired messenger for vaccine information. However, distrust linked to fear and misinformation and structural barriers to vaccine access persist.

## 5. Conclusions

Public health strategies to address the study results include health education on how vaccines are developed and identifying misinformation online; reinforcing medical providers’ communication skills in sharing vaccine information; and addressing social determinants of health. The focus group data highlight the distrust in vaccines, which has spiked since the COVID-19 pandemic, and how there is a need for this trust to be rebuilt. Public health efforts to increase vaccination confidence among rural, racially and ethnically diverse populations in the Southeast should address these factors using a hybrid approach that integrates both peer-led and provider-led messaging, along with multi-modal educational efforts. Peer-led and community-based interventions can effectively engage communities and reduce stigma, while provider-led messaging ensures the delivery of accurate and authoritative health information. For instance, peer leaders can initiate conversations and provide support, while healthcare providers can offer clinical guidance and address medical questions. The integration of technology (i.e., SMS, social media, and mobile apps) into these efforts can amplify the reach, uptake, and adherence among similar communities. These strategies would be most effective when embedded into the existing social infrastructure of communities.

## Figures and Tables

**Table 1:** Participant demographics *.

Demographic Variable	Community Members (*N* = 91) n/N (%)	Community Leaders * (*N* = 28) n/N (%)
**Race**		
White	8/87 (9)	2/27 (7)
Black or African American	73/87 (84)	17/27 (63)
American Indian or Alaskan Native	3/87(3)	1/27 (4)
Other	3/87 (3)	7/27 (26)
**Ethnicity**		
Hispanic or Latino	14/78 (18)	9/27 (33)
**Gender**		
Male	24/89 (27)	12/28 (43)
Female	62/89 (70)	14/28 (50)
Other	3/89 (3)	2/28 (7)
**Age Mean (SD)**	60 (15.2)	50 (26.1)
**Household Income**		
Less than USD 10,000	10/85 (12)	-
USD 10,000 to USD 29,999	35/85 (41)	-
USD 30,000 to USD 49,999	16/85 (19)	-
USD 50,000 to USD 69,999	9/85 (11)	-
USD 70,000 and above	6/85 (7)	-
Don’t know/Prefer not to answer	9/85 (11)	-
**Employment Status**		
Working full-time	18/86 (21)	-
Working part-time	9/86 (10)	-
Multiple part-time jobs	3/86 (3)	-
Not currently working	7/86 (8)	-
Retired	33/86 (38)	-
On disability	14/86 (16)	-
Other	2/86 (2)	-
**Level of Education**		
Less than high school	3/90 (3)	0/28 (0)
Some high school	16/90 (18)	0/28 (0)
High school diploma or GED	35/90 (39)	2/28 (7)
Some college/associate degree/trade school	26/90 (29)	8/28 (29)
College degree (bachelor’s)	9/90 (10)	10/28 (36)
Some graduate school	1/90 (1)	2/28 (7)
Graduate school	0/90 (0)	6/28 (21)
**Health Insurance**		
Private insurance	18/79 (23)	-
Medicare	30/79 (38)	-
Medicaid	13/79 (16)	-
Medicare and Medicaid (dual eligible)	16/79 (20)	-
Military insurance	2/79 (3)	-

* Not all focus group participants completed the survey or answered all questions on the survey when administered.

**Table 2:** Participants’ levels of trust in different groups *.

Please Rate Your Level of Trust with the Following Groups.	Not at All n/N (%)	Somewhat n/N (%)	A Lot n/N (%)	*p*-Value
How well do you trust the healthcare system?	3/109 (3)	52/109 (48)	54/109 (50)	<0.001
How well do you trust your church/spiritual leaders?	4/107 (4)	44/107 (41)	59/107 (55)	<0.001
How well do you trust leaders in your community?	3/105 (3)	70/105 (67)	32/105 (30)	0.002
How well do you trust healthcare providers?	1/106 (0.9)	42/106 (40)	60/106 (59)	<0.001
How well do you trust the media?	28/106 (26)	70/106 (66)	8/106 (8)	<0.001
How well do you trust the government?	34/106 (32)	64/106 (60)	8/106 (8)	<0.001

* Denominators may be different across items as not all focus group participants completed the survey or answered all questions on the survey when administered. Percentages may not add up to 100 due to rounding.

**Table 3:** Participants’ concerns about vaccines.

Which of the Following Concerns, if Any, Do You Have About Getting Vaccines?	Reported Concern *n* (%)
I don’t trust how vaccines were made and approved	23 (19%)
I would rather take the risk of getting sick with the disease than getting the vaccine	7 (6%)
I am worried vaccines may be harmful or have side effects	56 (47%)
I am worried there may be a cost with getting vaccines	22 (18%)
I don’t trust how vaccines have been given out to my community	13 (11%)
I am worried that I won’t have time to get vaccines	13 (11%)
With so many vaccines available, I’m worried about knowing which one is best for me	55 (46%)
I don’t have any concerns about getting vaccines	44 (37%)

## Data Availability

The participants of this study did not give written consent for their data to be shared publicly. Due to the sensitive nature of the study and the small sample size, the data are not available to be shared.

## Appendix A. Example of the Demographic and Attitudinal Survey

1. To which gender do you most identify?

____Male ____Female ____Other _____Prefer not to answer

2. What is your age? _______
3. What is your total yearly household income?

Less than $10,000	
$10,000 to $19,999	
$20,000 to $29,999	
$30,000 to $39,999	
$40,000 to $49,999	
$50,000 to $59,999	
$60,000 to $69,999	
$70,000 and above	
Don’t know	
Prefer not to answer	

4. Which of these groups best describes your race? (check all that apply)

White	
Black or African American	
American Indian or Alaskan Native	
Asian or Asian American	
Native Hawaiian or Other Pacific Islander	
Other, please specify:	

5. Would you describe yourself as being Hispanic or Latino?

_____Yes_____No

6. What is your employment status? Check all that apply.

Working Full time	
Working part time	
Multiple part time jobs	
Not currently working	
Retired	
On disability	
Other, please specify:	

7. What is the highest level of education you have completed?

Less than high school	
Some high school	
High school diploma or GED	
Some college/Associate degree/trade school	
College degree (bachelor’s)	
Some graduate school	
Graduate school	

8. What type of health insurance do you have?

Private Insurance	
Medicare	
Medicaid	
Medicare and Medicaid (Dual eligible)	
Military Insurance	

9. Please answer the following questions as strongly agree, somewhat agree, neither agree nor disagree, somewhat disagree, strongly disagree.

	Strongly agree	Somewhat agree	Neither agree nor disagree	Somewhat disagree	Strongly disagree
Vaccines work well in fighting against diseases.					
Vaccines are safe.					
The side effects of vaccines make me not want to get them.					
If someone already had a certain disease, they don’t need to get a vaccine because they are already protected.					
I would say I am hesitant when it comes to getting vaccines.					
When I think of getting vaccinated, I weigh benefits and risks to make the best decision possible.					

10. What vaccines have you received over the course of your life? Please check yes, no, or don’t know for each vaccine listed.

**Vaccine**	**Yes**	**No**	**Don’t Know**
Measles			
Mumps			
Rubella			
Tetanus			
Polio			
Hepatitis A			
Hepatitis B			
Human Papillomavirus (HPV)			
Flu vaccine			
Shingles			
Tuberculosis			
COVID-19			
Other: (please write in)			

11. Which of the following concerns, if any, do you have about getting vaccines? Check all that apply.

I don’t trust how vaccines were made and approved	
I would rather take the risk of getting sick with the disease than getting the vaccine	
I am worried vaccines may be harmful or have side effects	
I am worried there may be a cost with getting vaccines	
I don’t trust how vaccines have been given out to my community	
I am worried that I won’t have time to get vaccines	
With so many vaccines available, I’m worried about knowing which one is best for me	
I don’t have any concerns about getting vaccines	

12. Please rate your level of trust with the following groups.

	Not at all	Somewhat	A lot
How well do you trust the healthcare system?			
How well do you trust your church/spiritual leaders?			
How well do you trust leaders in your community?			
How well do you trust healthcare providers?			
How well do you trust the media?			
How well do you trust the government?			

13. Which of the following sources do you prefer to get vaccine-related information from? Check all that apply.

Television/radio/podcasts	
Social media	
Spouse/partner and other family members	
Friends or co-worker	
Religious leader	
Celebrities	
Doctor/medical provider	
Newspaper (printed or online)	
Government or another official website	

14. Which of the following news sources do you watch or listen to, to get vaccine information? Check all that apply.

CNN	
MSNBC	
NBC	
CBS/ABC	
NPR	
Other national networks. Please specify:	
Other international networks. Please specify:	

## Appendix B

**Table A1:** Similarities across community member and community leader descriptive quotes by theme.

Theme	Community Leader Quote	Community Member Quote
Theme 1: Differences in trust based on how long a vaccine has been available	“My concern was it was something new… I had to decide whether I wanted to trust getting it in a shot… So, you know what I did, took the shot.”	“I just don’t trust none of them for real because just like after COVID came out… How do you know this medicine’s going to work?”
Theme 2: Distrust influenced by misinformation, often spread through social media	“All these horrible imaginations started spreading on social media, and it didn’t help it became political.”	“I think social media… makes it worse… they tell us stuff that’s not true.”
Theme 3: Concern about getting sick after a vaccine	“I’ve had bad situations with the flu. It was like I’d rather have the flu than the vaccine.”	“I got the flu the same day. My body was aching. I was sick… I was sick as a dog.”
Theme 4: Trusting vaccines due to seeing the impact of not taking them	“So many people were dying from it… I said, Lord, please send something… I’m ready for whatever to happen.”	“Back in the day… people were getting like measles… they were dying because they didn’t have it… but now they’re not dying.”
Theme 5: Trusted leaders advise people to do their own research on vaccines	“I checked how it was made and who made it. I was real impressed with Moderna.”	“I went on the computer and checked things out…That’s why I trusted Moderna.”
Theme 6: Trust in messages from medical providers	“If you’re a doctor and servicing me, I gotta have some trust in him.”	“You could ask whatever you wanted to ask to your doctor, and he would break it down to you.”
Theme 7: Social determinant barriers to vaccination (cost, education, transportation)	“A lot of older people couldn’t get it because they don’t have transportation.”	“I didn’t have any money to give anybody for a ride.”
